# The effects of radiofrequency exposure on male fertility and adverse reproductive outcomes: A protocol for two systematic reviews of human observational studies with meta-analysis

**DOI:** 10.1016/j.envint.2021.106968

**Published:** 2022-01

**Authors:** Ryan P.W. Kenny, Evelyn Barron Millar, Adenike Adesanya, Catherine Richmond, Fiona Beyer, Carolina Calderon, Judith Rankin, Mireille Toledano, Maria Feychting, Mark S Pearce, Dawn Craig, Fiona Pearson

**Affiliations:** a Evidence Synthesis Group, Population Health Sciences Institute, Newcastle University, UK; b Maternal & Child Health Group, Population Health Sciences Institute, Newcastle University, UK; c UK Health Security Agency, Chilton, Didcot, UK; d Mohn Centre, Imperial College London, UK; e Karolinska Institutet, Sweden

**Keywords:** Radiofrequency exposure, Electromagnetic fields, Non-ionizing radiation, Fertility, Pregnancy outcomes

## Abstract

**Background:**

The World Health Organization (WHO) is bringing together evidence on radiofrequency electromagnetic field (RF-EMF) exposure in relation to health outcomes, previously identified as priorities for evaluation by experts in the field, to inform exposure guidelines. A suite of systematic reviews are being undertaken by a network of topic experts and methodologists in order to collect, assess and synthesise data relevant to these guidelines. Here, we present the protocol for the systematic review on the effect of exposure to RF on adverse reproductive outcomes (human observational studies), also referred to as Systematic Review (SR) 3 within the series of systematic reviews currently being commissioned.

**Objectives:**

Following the WHO handbook for guideline development and the COSTER conduct guidelines, we will systematically review the effect of RF-EMF exposure on both male fertility (SR3A) and adverse pregnancy outcomes (SR3B) in human observational studies. Herein we adhere to the PRISMA-P reporting guidelines.

**Data sources:**

We will conduct a broad search for potentially relevant records relevant for both reviews within the following bibliographic databases: MEDLINE; Embase; and EMF Portal. We will also conduct searches of grey literature through relevant databases and organisational websites. RF-EMF experts will also be consulted. We will hand search citation and reference lists of included study records.

**Study eligibility criteria:**

We will include quantitative human observational studies on the effect of RF-EMF exposure: (in SR3A) in adult male participants on infertility, sperm morphology, concentration or total sperm count or motility; and (in SR3B) in preconception adults or pregnant women on preterm birth, small for gestational age (associated with intrauterine growth restriction), miscarriage, stillbirth and congenital anomalies.

**Study appraisal and synthesis methods:**

Titles, abstracts and then full texts will be screened in blinded duplicate against eligibility criteria with input from a third reviewer as required. Data extraction from included studies will be completed by two reviewers as will risk of bias assessment using the Office of Health Assessment and Translation (OHAT) tool. If appropriate we will undertake meta-analysis to pool effect measures and explore heterogeneity using sub-group analyses or meta-regression as feasible. We will conduct sensitivity analysis to assess the impact of any assumptions made throughout the review process. The OHAT methodology, based on the GRADE guidelines for evidence assessment, will be used to evaluate the certainty of evidence per outcome and to conclude the level of evidence of a health effect.

**Conclusion:**

This manuscript details the protocols for two systematic reviews. The aims of publishing details of both protocols are to: pre-specify their scope and methods; reduce the impact of reviewer bias; promote transparency and replicability; and improve the review process.

**Prospero registration:**

CRD42021265401 (SR3A), CRD42021266268 (SR3B).

## Graphical abstract

**Figure:**
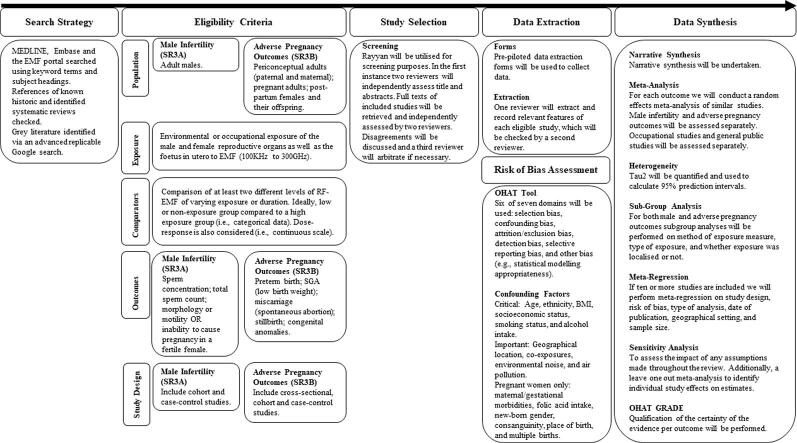

## 1 Introduction

### 1.1 Background

The technological applications of radiofrequency electromagnetic fields (RF-EMF; frequencies 100 kHz–300 GHz) have been steadily increasing since the 1950s. RF-EMF are used in medicine (e.g. magnetic resonance imaging, diathermy, radiofrequency ablation), industry (e.g. heating and welding), domestic appliances (e.g. baby monitors, Wi-Fi), security and navigation (e.g. radar and radio frequency identification; RFID) and especially in telecommunications (e.g. radio and TV broadcasting, mobile telephony). These developments mean that large parts of the global population are now exposed to an increasing range of RF-EMF sources over increasing durations. Concern has been raised regarding the public health consequences from exposure to RF-EMF and it is therefore crucial to perform a health risk assessment to inform exposure guidelines.

The World Health Organization (WHO) has an ongoing project to assess potential health effects of exposure to RF-EMF in the general and working population. To prioritise the assessments of potential adverse health outcomes from exposure to these fields, the WHO conducted a broad international survey amongst RF experts in 2018 (Verbeek et al., 2021). Six priority topics were identified (cancer, adverse reproductive outcomes, cognitive impairment, symptoms, oxidative stress, and heat related effects). The WHO subsequently commissioned ten systematic reviews of observational and experimental studies to collect, assess and synthesise the available evidence on these topics.

### 1.2 Description of the exposure

RF-EMF fields are generated by many devices used both within the living and working environments. Sources kept close to the body, such as mobile phones and Bluetooth headsets, will result in very localised exposure, whereas sources far away from the body, such as base stations used for communication (mobile network, TV and radio), will result in a more uniform exposure across the whole body. Electromagnetic fields are described by their amplitude (electric field strength, magnetic field strength, or power density), their frequency, spatial distribution and temporal variation (for pulsed or discontinuous sources).

RF-EMF does not have enough energy to cause ionisation in matter, but it may be absorbed by the human body. The only established adverse health effect from RF-EMF is the heating of tissues above 41 °C. Consequently, current exposure guidelines protect the reproductive organs and foetus against tissue temperature rises above 2 °C (International Commission on Non-Ionizing Radiation Protection (ICNIRP), 2020), by setting basic restrictions on the rate of energy absorbed by tissues, the Specific Absorption Rate (SAR). EMF sources in the environment typically result in exposure levels well below these restrictions, although occupational exposures can be higher (Advisory Group on Non-Ionising Radiation, 2003, SCIENTIFIC COMMITTEE ON EMERGING AND NEWLY IDENTIFIED HEALTH RISKS (SCENIHR), 2015, McKinlay et al., 2004). SAR depends on the RF-EMF parameters described above, as well as the dielectric properties of the human body, the shape and position of the body with respect to the source and the grounding conditions. It is a quantity which is complex to calculate and cannot be measured within the human body. Thus, exposure is often described in terms of the field characteristics.

### 1.3 Description of the outcome

The term “adverse reproductive outcomes” encompasses a heterogenous set of endpoints (from a clinical perspective): inability to conceive which occurs in 15% of couples with up to 50% being due to male infertility factors and “adverse pregnancy outcomes” such as spontaneous miscarriage which occurs in 25% of pregnancies (Wang et al., 2021); pre-term birth occurring in 10% of pregnancies; stillbirth occurring in 2% of births; congenital anomalies occurring in up to 5% of newborns and low birth weight occurring in 14.6% of births (Blencowe et al., 2019).

These reproductive outcomes are linked with other detrimental events over the lifecourse, impacting health beyond their own occurrence. Spontaneous miscarriage is indicative of premature mortality in mothers (before age 70), particularly increased risk of death from cardiovascular disease (Wang et al., 2021). Preterm birth, especially in very early stages, is a serious condition that can lead to life-long complications for the child. For example, babies born before 37 weeks’ gestation are at a higher risk of neurodevelopmental disorders and respiratory and gastrointestinal impairments (Etzel, 2020). Intrauterine growth restriction may be associated with preterm birth through medically indicated preterm induction of labour but may also carry separate health consequences. Intrauterine growth is indicated by small for gestational age (SGA) or low birth weight adjusted for gestational age (Sharma et al., 2016). A low birth weight has been reported as an important predictor of morbidity and mortality in neonates, childhood, and adults (Lee et al., 2020). Epidemiological evidence demonstrates that environmental exposures can influence fetal growth and development via induction of changes in fetal growth patterns (Lee et al., 2020).

Male infertility is defined as the inability of a man to cause pregnancy in a fertile female after 12 months or more of regular unprotected sexual intercourse (World Health Organization, 2020a). Such infertility is strongly correlated with a lack of viable spermatozoa (e.g. reduced sperm concentration or total sperm count; (Levine et al., 2017)). Declining sperm concentration has been consistently reported, and debated, over the past fifty years (Levine et al., 2017), attributed to a range of environmental and lifestyle exposures, such as pesticides, endocrine disrupters, body mass index (BMI), or type II diabetes.

Abnormal sperm morphology can also affect a man’s ability to cause pregnancy, especially when abnormal morphologies occur in high quantities. Additionally, abnormally shaped sperm are usually associated with other semen irregularities such as low sperm count or motility (American Society for Reproductive Medicine, 2014). Healthy sperm motility has forward progressions of at least 25 µm per second, containing at least 50% grade A and B progressively motile sperm. If these factors are not met the sperm may have difficulty passing though the cervical mucus, leading to failure in fertilization (Kumar and Singh, 2015).

### 1.4 Rationale for this systematic review

In 2018, a survey amongst RF-EMF experts was performed by the WHO to prioritise potential adverse health effects for investigation. Survey results showed that 32% of respondents deemed adverse pregnancy outcomes as critical for decision making. Regarding effects on male fertility, 28% of respondents deemed it critical for decision making. As such, these outcomes were indicated for further investigation (Verbeek et al., 2021). The reasoning given by the respondents (RF-EMF experts) for applying these ratings were public concern, knowledge from animal studies, from human studies and burden of disease.

Several mechanisms have been theorised that could cause RF-EMF exposure to lead to adverse health effects including adverse reproductive outcomes but none, so far, have been validated. These theorised mechanisms include: oxidative stress caused by the radical pair mechanism or changes to free radical homeostasis; changes in magnetite particle activity influencing cellular processes; non-equilibrium (e.g. thermal equilibrium) and non-linear effects (e.g. demodulation); changes to Ca^2+^ ion homeostasis; pearl chain formation, whereby molecules and cells move towards the direction of the electric field; and (most commonly accepted) causing a thermic/heating action at a microscopic level (Scientific Committee on Emerging and Newly Identified Health Risks (SCENIHR), 2015).

There are published studies showing that exposure to RF-EMF could have a detrimental effect on pregnancy outcomes (e.g. miscarriage, congenital malformations, low birth weight, and preterm birth (Shah and Farrow, 2014, Mahmoudabadi et al., 2015, Kesari et al., 2018)). Additionally, evidence exists indicating that male reproductive outcomes (e.g. sperm motility, morphology, viability, and concentration) could potentially be affected by RF-EMF exposure (Kesari et al., 2018). For example, a review combining in-vivo and in-vitro evidence suggests mobile phone use negatively impacts sperm quality (Adams et al., 2014). However, there is also evidence suggesting that mobile phone use does not negatively impact birth weight or foetal growth (Tsarna et al., 2019). Narrative reviews have been performed to assess the evidence on RF-EMF regarding potential adverse health effects (INTERNATIONAL COMMISSION ON NON-IONIZING RADIATION PROTECTION (ICNIRP), 2020, Advisory Group on Non-Ionising Radiation, 2003, SCIENTIFIC COMMITTEE ON EMERGING AND NEWLY IDENTIFIED HEALTH RISKS (SCENIHR), 2015). Overall, the evidence to date on RF-EMF exposure and adverse reproductive outcomes is unclear.

Systematic reviews adhere to strict scientific design based on pre-defined, explicit, and reproducible methodology (Gopalakrishnan and Ganeshkumar, 2013). As such, their findings are generally less biased with more certainty than those from narrative reviews. To our knowledge, there is no existing systematic review encompassing articles assessing the effect of multiple sources of RF-EMF in general living and work environments on both male and female reproductive outcomes.

## 2 Objectives

In the current paper, we present the protocols for two systematic reviews of human observational studies on exposure to RF-EMF and: male infertility (SR3A); or adverse pregnancy outcomes (SR3B). The primary objectives of both reviews are to address the following (Population/Exposure/Comparator/Outcome; PECO) questions:

• What are the effects of localised and whole-body RF-EMF exposure (E) on male infertility; sperm morphology; motility; concentration or count, and time to pregnancy (O) compared to no/low level of exposure (C) in adult males (P) within human observational studies?
• What are the effects of localised and whole-body RF-EMF exposure (E) on preterm birth; SGA; miscarriage; still birth and/or congenital anomalies (O) compared to no/low level of exposure (C) in preconception or pregnant adults (P) within human observational studies?

A secondary objective is to assess whether an exposure dose–response relationship between the RF-EMF exposure and adverse reproductive outcomes exists.

## 3 Methods

**Fig. 1:**
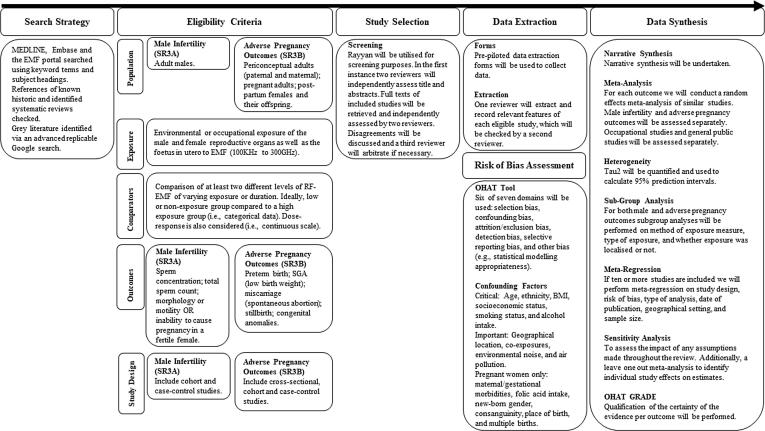
An overview of the systematic review process.

The protocols for the reviews have been registered in PROSPERO under CRD42021265401 (SR3A) and CRD42021266268 (SR3B). An overview of the protocol can be observed in Fig. 1. This protocol has been designed so the review adheres with the Conduct of Systematic Reviews in Toxicology and Environmental Health Research (COSTER) guidelines (Whaley et al., 2020) and adheres to the preferred reporting items for systematic review and meta-analysis protocols statement (PRISMA-P; See Supplementary File 1; (Moher et al., 2015)) and the WHO Handbook for Guideline Development (World Health Organization, 2014). In case any amendments to this protocol are made during the review process, changes and related reasons will be reported in the final article.

### 3.1 Eligibility criteria

The PECO criteria (Morgan et al., 2018) are described below. The criteria and hierarchy of use during screening is given within Supplementary File 2.

For those records deemed eligible for inclusion the methods of exposure and outcome assessment used will be recorded during data extraction, evaluated during risk of bias assessment (Supplementary Files 3 and 4) and any potential impact due to the exposure assessment method used will be explored in analyses (e.g. misclassification, response/behaviour bias, measurement error).

#### 3.1.1 Populations

In SR3A, we will consider for inclusion studies reporting on the influence of environmental RF-EMF exposure to adult male participants on infertility outcomes.

In SR3B, we will consider for inclusion studies reporting on the influence of RF-EMF exposure to periconceptual adults (paternal and maternal) or pregnant adults on adverse pregnancy outcomes. As such, we will also consider for inclusion studies including post-partum females and their offspring.

#### 3.1.2 Exposures

Environmental exposure of the male and female reproductive organs as well as the foetus in utero can result from whole-body or more localised exposure. We must consider the specificity of the exposure and proximity to the reproductive organs (e.g. a phone call vs internet browsing with a phone on a lap). In terms of public exposure, whole body (uniform) exposure can result from, for example, exposure emitted by radio- and television masts, and mobile phone base stations. These exposures are continuous low level exposures, typically below 1 V/m (Gajšek et al., 2015). More localised exposure can result from, for example, keeping a mobile phone in the pocket of the trousers close to the reproductive organs while transmitting (e.g. using hands-free kit during a call) or using Wi-Fi on a laptop placed on the lap. These exposures are, however, intermittent and decay sharply with distance. In between these two types of exposures are sources in the nearby environment such as digital enhanced cordless telecommunications (DECT) base stations, wireless local area network (WLAN) access points, femtocells, baby monitors and smart meters. These sources result in intermittent inhomogeneous whole or partial body exposure and are typically low powered (<1 W), and thus result in low exposures. SR3A and SR3B will include studies of the effect of both whole-body and more localised exposure. Whilst our interest is in RF-EMF exposure to the reproductive organs, it is most likely that evidence will report upon the effect of whole-body exposure. It is common for exposure from different sources (e.g. mobile phones, masts, WLAN, digital home phones) to be combined during assessment in primary studies (e.g. using broadband personal exposure meters), with RF-EMF assessed at the whole-body level. If data are available based on whole-body and localised exposure to the reproductive organs, where plausible we will analyse these data separately using appropriate subgroup analysis, although we anticipate this type of analysis as being highly unlikely. Typically, the public is exposed to whole body RF-EMF, which occurs at a low level compared to more localised sources that would directly affect the reproductive organs. As such, the whole-body exposure route differs from localised exposure and we will consider this in the GRADE assessment when assessing indirectness. Specifically, we will consider whether downgrading is needed for whole body exposure assessments compared to localised.

Specific absorption rate (SAR), expressed in watts per kilogram (W/kg) would be the ideal exposure measurement of interest for both SR3A and SR3B. It is unlikely, however, that SAR at the reproductive organs will be readily provided, and even if the publications had some information that would enable the rough estimation of dose at the organ level (Liorni et al., 2020), this estimate would be subject to large uncertainties, and may not be adequate except for perhaps differentiating between highly exposed groups. We will thus also include epidemiological studies using surrogate RF-EMF exposure measures that rely on measured or modelled levels of electric or magnetic fields or power density (e.g. at the participants residence) or on exposure proxies as mentioned below.

One of the most used technologies emitting RF-EMF are mobile phones, which have been available since 1984 worldwide and since 1979 in Japan. We expect a large proportion of studies identified to be investigating this exposure source. Over the decades, the systems used for mobile communication have evolved, each with their own RF exposure characteristics, in terms of frequency and average transmitting power. For studies of mobile phone use, exposure assessments may be based on self-reporting of proxy measures of exposure such as hours of use. We will include studies with both objective phone use measurement and self-reported phone use because these measurements are known to be well correlated, although this varies depending on the outcome measure and age (VANDEN ABEELE, M., BEULLENS, K. & ROE, K., 2013, Samkange-Zeeb et al., 2004).

Base stations are also a common environmental source of RF-EMF exposure. For this exposure source, we will only include studies in the systematic review utilising objectively measured distance to source assessments (e.g. derived from geocodes) (Martens et al., 2017). Studies utilising self-reported distance to source assessments will be excluded as self-report measures are not well correlated with actual measures (Martens et al., 2017). When identifying distance to source estimates, special care must be taken in scenarios where multiple different transmitters are included in the same study (Schmiedel et al., 2009). It must also be borne in mind that distance from a base station may be a poor indicator of exposure to RF-EMF when in indoors, due to the complex propagation characteristics of emissions from base station antennas (such as shielding and multiple reflection effects; (Frei et al., 2010)).

If identified, studies utilising spot measurements, personal exposimeters and prediction models will be included.

We also expect a large proportion of studies to be investigating occupational exposure sources. Occupational RF exposure occurs from a multitude of sources, such as navigation systems, broadcast and telecommunication equipment, security and access controls, plasma discharge equipment, tape erasers, welding equipment, and radar (Advisory group on Non-ionising radiation, 2003). Exposure levels vary dramatically across and within jobs where equipment or device use/operation and maintenance occur (Hareuveny et al., 2015).

Occupational exposure information can be based upon measurements, observations, expert assessment or combinations of these (Bondo Petersen et al., 2018). We will include studies that have measured exposure to RF-EMF at work using any of the aforementioned methods or when an exposure level is modelled based on job-exposure matrices (JEMs), but not when this is done based on job title alone. JEMs are occupational exposure assessment tools based on cross-tabulations of occupations with exposure data for a well-defined occupational exposure in a given time window and geographical area where probability and intensity have been scored by exposure experts.

Where exposure assessment is reported as a dichotomous answer to a question indicating exposure to a source vs none (e.g. ‘*have you ever owned a mobile phone?’* yes/no) studies will not be eligible for inclusion due to the high level of imprecision in this approach.

Studies of exposure from medical technologies will be excluded if the population of interest are patients rather than workers using the technology and exposed occupationally.

When evaluating the outcomes of interest, paternal exposure of the testes during the three months prior to pregnancy is of most interest. When evaluating the effect of maternal exposure on the outcome preterm birth, exposure during the whole pregnancy will be considered first, then the first and second trimesters. When evaluating the outcome SGA and congenital anomalies, we will consider exposure across the whole pregnancy. Where possible we will consider each trimester individually. Timing of exposure will not be used as an exclusion criterion but will be considered in risk of bias assessments (Bonde et al., 2019, Anand-Ivell et al., 2018, Selevan et al., 2000, Cohen, 2014, Wigle et al., 2007, Porpora et al., 2019).

#### 3.1.3 Comparators

Studies will ideally compare RF-EMF exposure in either a low exposure or non-exposure group to a ‘high’ exposure group (i.e. categorical data). However, as guidelines exist dictating acceptable environmental and occupational RF-EMF exposure levels it is likely that ‘high’ exposure groups will fall below these bounds. Given this, studies which have compared at least two different levels of RF-EMF of varying exposure or duration will also be considered. Additionally, if the studies present dose–response data with a continuous scale of varying RF-EMF exposure they will also be included.

#### 3.1.4 Outcomes

##### 3.1.4.1 Male fertility outcomes (SR3A)

Infertility is defined, by the WHO (2020), as a reproductive system disease denoted by the inability to achieve a clinical pregnancy after 12 months or more of regular unprotected sexual intercourse (World Health Organization, 2020a). We will include dichotomous assessments of fertility as well assessments of time to pregnancy.

Newly diagnosed cases of male infertility, based on a physician’s diagnosis and in agreement with the definition of the inability to cause pregnancy in a fertile female after a period of follow-up, will be included. Studies which assess sperm concentration or total sperm count, morphology or motility will also be included if completed objectively (using an expert evaluator adequately blinded when appropriate) in a quantitative manner. Studies of more specific sperm parameters will be excluded as the validity of such diagnostic methods for infertility has not been established.

We will include both categorical and continuous assessments of sperm concentration and total sperm count, sperm morphology and motility. The WHO reference ranges will be used to establish normal values.

Studies using self-reported outcomes of male infertility will be excluded.

##### 3.1.4.2 Adverse pregnancy outcomes (SR3B)

The outcomes of interest are preterm birth, SGA (including low birth weight at term as indicators of intrauterine growth restriction), miscarriage (sometimes termed spontaneous abortion), stillbirth and congenital anomalies.

Preterm birth is defined as being born before 37 completed weeks of gestation. The following categories of preterm birth will be used: very preterm as born before week 32 and after week 28; and as extreme preterm as born before week 28, diagnosed by any measure (e.g. date of last menstrual period, based on ultra-sound as assessed by a health care professional such as a midwife or a physician, or extracted from medical records or data registers). The definition of preterm birth is likely to vary depending on the geographic setting of the study. We will collect data on preterm birth, alongside author definitions, and perform a sensitivity analysis if appropriate.

SGA can be reported as intrauterine growth restriction or low birth weight (Suhag and Berghella, 2013). SGA is defined as birth weight below the 10th percentile for newborns of the same gestational age (based on assessment by a health care professional or extracted from medical records or health data registers). Low birth weight will be identified using WHO reference ranges for normal values in specific settings (Schlaudecker et al., 2017, World Health Organization, 2004).

Miscarriage, will be defined as pregnancy loss before 24 weeks that is not associated with medical or surgical intervention to terminate the pregnancy, based on assessment by a health care professional or extracted from medical records or data registers. The definition of miscarriage also varies depending on geographic setting, for example in the USA, it is defined as pregnancy loss before 20 weeks (Centers for Disease Control and Prevention, 2020). We will collect data on miscarriage, alongside author definitions, and perform a sensitivity analysis if appropriate.

Stillbirth is defined (in the UK) as non-live birth after 24 completed weeks of pregnancy (World Health Organization, 2020b) based on an assessment by a health care professional or extracted from medical records or data registers will be considered. As follows the definition of stillbirth also varies depending on geographic setting, in the USA aligned to the definition of miscarriage, it is non-live birth after 20 weeks pregnancy (Centers for Disease Control and Prevention, 2020). As for miscarriage and pre-term birth, we will collect data on stillbirth, alongside author definitions, and perform a sensitivity analysis if appropriate.

Congenital anomalies, defined as structural or functional abnormalities (e.g. metabolic disorders) that are present from birth (World Health Organization, 2010), will be subdivided according to organ system. Studies which measure congenital anomalies based on an assessment by a health care professional or extracted from medical records or data registers will be considered.

Studies using self-reported outcomes of preterm birth, SGA (including low birth weight at term), miscarriage, stillbirth and congenital anomalies will be excluded.

#### 3.1.5 Types of studies

##### 3.1.5.1 Inclusion criteria

###### 3.1.5.1.1 Male fertility outcomes (SR3A)

We will consider cohort and case-control studies to be eligible for inclusion. A cohort study is usually defined as a study where there are two or more groups exposed to different levels of RF-EMF or no exposure and that are followed over time to assess the occurrence of infertility or adverse sperm parameters. Additionally, we will consider studies if the analysis is conducted using dose–response methods. Case-control studies are defined as studies in which previous exposure to RF-EMF in infertile men, the cases, is compared to the exposure in fertile controls.

Cross-sectional studies of male infertility will be excluded because of the lack of temporality in these studies which makes it difficult to establish causal effects.

###### 3.1.5.1.2 Adverse pregnancy outcomes (SR3B)

We will include cross-sectional, cohort, and case-control study designs. Commonly, pregnancy outcomes studies are cross-sectional (i.e. they study the prevalence of the outcome at birth but cannot determine when during the pregnancy the outcome occurred). However, studies can also utilise a cohort or case-control study design. In this review, it is more relevant to focus on the timing of the collection of the exposure information. Cohort studies are often completed in a prospective manner, although in some cases they can be retrospective or at least contain a retrospective component. Case-control studies are always retrospective. Additionally, we will consider studies if the analysis is conducted using dose–response methods. In retrospective studies of pregnancy outcomes, the population of the study may be post-partum women, when these are assessing the impact of RF-EMF exposure pre-conception or during pregnancy they will be included.

##### 3.1.5.2 Exclusion criteria

Case reports, pre-clinical and in vitro studies will be excluded. Studies with self-selection of participants from an unidentified study population, e.g. through advertisement, will be excluded.

##### 3.1.5.3 Years considered

Searches will be conducted from inception of the databases and we will not place any restrictions on year of publication.

##### 3.1.5.4 Publication language

We will include studies written in any language, provided that an English translation can be obtained.

##### 3.1.5.5 Publication types

We will include quantitative studies reported in the research literature.

#### 3.1.6 Types of effect measures

For dichotomous outcomes, relative risk (RR) will be used as the measure of the effect of one unit of exposure compared to one lower-level unit of exposure. Odds ratios (OR) and hazard ratios (HR) will also be used where appropriate. These will be considered similar unless the occurrence of the outcome of interest is more than 10%. Where the incidence of the outcomes of interest is not low, all effect sizes will be transformed into RRs using the appropriate calculations. For continuous outcomes, mean differences (MD) will be used. When the same outcome is measured using different scales standardised mean difference (SMDs) will be calculated.

### 3.2 Information sources and search strategy

Eligible studies will be identified by literature searches through MEDLINE and Embase. The EMF Portal, a dedicated database of the scientific literature on the health effects of exposure to electromagnetic fields (https://www.emf-portal.org/en) will also be consulted. The search strategy has been developed iteratively based upon concepts integral to each review question and incorporates up to date keyword terms and subject headings as well as outcome measures identified by clinical experts (See Supplementary File 2).

These searches will be supplemented by checks of the reference lists of previous systematic reviews, as far as such reviews are available. Reference checking will also be carried out on included studies as will citation checking. Papers highlighted by topic experts will also be evaluated for inclusion.

No language or date restrictions will be applied to the search. The search results will be exported into Endnote and duplicates removed before screening commences.

Grey literature will be identified, focusing on guidelines and reports from public health and radiation protection bodies, theses and EMF conferences. Web of Science (conference abstracts) and IEEE Xplore® will be searched to identify grey literature of relevance. An internet search using advanced search functionality in Google, and other search engines if appropriate, will also be conducted.

### 3.3 Study selection

De-duplicated search results will be exported from EndNote to Rayyan (Ouzzani et al., 2016) for screening. Rayyan is a web-based application that was designed to speed up the process of screening and study selection. Two reviewers will independently check the relevance of the identified papers based on titles and abstracts. We will exclude irrelevant records that certainly do not fulfil at least one of the inclusion criteria. Full texts of records included at this stage will be sourced. Two reviewers will then independently assess included records based on full texts. This will result in a final list of included and excluded studies. If findings from a study are reported in more than one article, we will consider all these papers together as one study. In this case, we will use the original study (i.e. the first publication). We will only extract findings reported in subsequent articles when relevant and not already available from the original publication. Across all steps, disagreements between the reviewers will be resolved by discussion. A third reviewer will be consulted if no consensus can be reached. We will document the selection process in a study flow diagram according to PRISMA reporting guidelines (Liberati et al., 2009).

The results from grey literature searching will be carefully evaluated by a single reviewer.

### 3.4 Data extraction

For both reviews, a standard set of details will be extracted from the relevant publication(s). The data relevant for the epidemiological studies include:

• first author and publication year, full reference;
• design (cohort; case-control);
• location (country, region, state, etc.);
• dates of study and sampling time frame (period of case ascertainment);
• demographics (age, occupation, SES, pregnancy duration at inclusion);
• number of subjects eligible, participation and follow-up rates;
• baseline differences;
• definition and measurement of the outcome;
• inclusion/exclusion criteria, and recruitment strategy.
• for case-control studies, definition of cases and controls (population based (incidence density sampling, or other method), hospital based (type of diagnoses), other types of controls);
• exposure source (when known)
• exposure assessment:
  o Public: measurements, self-administered questionnaire, personal interview; computer assisted personal interview; register based sources;
  o Occupational: measurements (PEM, spot), exposure based on JEM, company records, questionnaires (self-administered, personal)
• exposure variables used in the analyses;
• effect size and 95% confidence intervals both from unadjusted and most adjusted models per exposure category, or per exposure increment;
• confounders and how considered in analysis, and
• funding source.

Based on mutually agreed piloted excel forms for data extraction (See Supplementary File 3), one reviewer will extract and record the relevant features of each eligible study. A second reviewer will check the extracted study information against the accompanying article(s) for completeness and accuracy and using the excel comments feature. The reviewers will resolve any possible disagreements by discussion; a third reviewer will be involved to resolve any conflicts. For continuous and categorical data, we will extract all available data regarding exposure. When exposure is reported on a continuous scale an estimated median value will then be assigned, as proposed by (Il’yasova et al., 2005). Whilst For studies that report the exposure in categories a single exposure value will be assigned to each category: for closed categories, the midrange score will be used; and for the (uppermost) open-ended categories, a value based on the lower bound and the width of the previous (second-to-highest) interval will be calculated (Il’yasova et al., 2005).

Studies combining original data from a set of primary studies in pooled analyses are a special case of multiple publications per study and may include either the whole primary datasets (completely overlapping pooled analyses), or a subset of individual data from the primary studies, plus additional unpublished data (partially overlapping pooled analyses). Pooled analyses of primary studies are eligible for inclusion if they include a comprehensive set of data previously published in individual primary studies. If assumptions about overlap of data are required, sensitivity analysis will be used to test robustness of the findings to changes in the dataset composition.

#### 3.4.1 Dealing with missing data

In the case that data necessary for the analysis are missing from published studies, authors of articles published in the last ten years will be contacted. In case of no response (within two weeks of initial contact), where feasible we will attempt to impute data based on other available data items.

### 3.5 Risk of bias assessment

#### 3.5.1 Risk of bias in studies

For both reviews, the risk of bias assessment will be conducted at study and outcome level using the “Risk of Bias Rating Tool for Human and Animal Studies” developed by the National Toxicology Program Office of Health Assessment and Translation (OFFICE OF HEALTH ASSESSMENT AND TRANSLATION (OHAT). , 2015, Rooney et al., 2014). Seven domains will be assessed: selection/participation bias; exposure measurement errors; inaccurate outcome assessment; uncontrolled confounding; incomplete outcome assessment due to attrition/exclusion; selective outcome reporting; and other potential threats to internal validity. Each domain is rated with one of four options: definitely low, probably low, probably high, and definitely high risk of bias (See Supplementary Files 3 and 4). If one of the authors of the review is also an author of an included study, we will make sure that this author will not extract data from their own study and will not judge the risk of bias. Assessments will be documented within mutually agreed piloted excel forms using the excel comments feature as required (See Supplementary File 3).

Factors associated with preterm birth, SGA or low birth weight or birth weight adjusted for gestational age (indicators of intrauterine growth restriction), miscarriage, stillbirth, congenital anomalies, and factors associated with male infertility and RF-EMF exposure will be taken into account as potential confounders. Confounders will vary between male, pregnancy, and offspring outcomes. The following critical confounder relationships have been identified by experts in the RF-EMF field and will be assessed for both SR3A and SR3B: age, ethnicity, BMI, socioeconomic status (SES) smoking status and alcohol intake. The following confounders are considered important but not critical; geographical location, co-exposures (e.g. occupation exposure to hazardous substances and heat), environmental noise and air pollution. Confounders that may only effect pregnant women and are considered important but not critical include maternal or gestational morbidities, folic acid intake, new-born gender, consanguinity, place of birth (setting), and whether the pregnancy is a multiple. Lack of confounding control will not be a reason for exclusion but will be considered in risk of bias assessments. Any further confounders highlighted during data extraction will be carefully considered.

For exposure, it must be emphasised that population average whole-body absorption is dominated by communication devices used close to the body (e.g. mobile and cordless phones, tablets) and not by far field sources such as base stations. Therefore, risk of bias analyses must consider whether exposure from these sources is being considered in a meaningful manner, either by adjusting or by restricting to time-periods when use of devices was in proximity to reproductive organs. Other errors in exposure estimates may occur but are less likely and perhaps harder to discern. For example; an inability to identify the true output power of mobile phones during calls because of power minimising when a base station signal level is high or vice versa (adaptive power control); impact of various different communication systems being used at the same time; an inability to estimate accurate tissue level exposure without records of the frequency a phone uses.

#### 3.5.2 Assessment of reporting biases

To assess publication bias we will create funnel plots and visually inspect them for missing small studies. Egger’s test will be used for categorical outcomes and we will use the method proposed by (Doleman et al., 2020) for continuous outcomes. Briefly, baseline risk is included as a study-level covariate (x-axis) and the observed asymmetry (considering meta-regression residuals) as the outcome, rather than mean difference, and the inverse sample size as the exploratory variable (instead of standard error; y-axis (Doleman et al., 2020). For dichotomous outcomes the arcsine test will be utilised (Rücker et al., 2008).

Selective outcome reporting bias will be considered in risk of bias assessment which is then considered in OHAT methodology (Office of Health Assessment and Translation (OHAT), 2015), based on the GRADE guidelines for evidence assessment, to evaluate the certainty in evidence of a health effect(Guyatt et al., 2011, Rooney et al., 2014).

### 3.6 Synthesis of results

We will follow the approach to conducting narrative synthesis as outlined by (Popay et al., 2006) on behalf of the UK’s Economic and Social Research Council methods programme. For each outcome of interest, we will conduct a meta-analysis of similar studies with a random effects model in R or similar software. Meta-analyses of incremental RRs will be performed using the generalised least squares for trend estimation of summarised dose–response (glst) method (Orsini et al., 2006, Orsini et al., 2012). If sufficient data for this analysis are not available, we will conduct a random effects meta-analysis of incremental RRs based on the general inverse variance method.

The mean of the combined effect sizes will be calculated in studies where several effect sizes were reported from the same sample (e.g., models with different control variables). An overall estimate will be calculated for studies with overlapping samples. In studies reporting effect sizes from independent subgroups (e.g., moderators), each subgroup will be included as a unique sample in the meta-analysis.

We will model the exposure in different ways where plausible. First, we will base the exposure contrast on differences in exposure intensity and then according to the duration of the exposure. We will also compare the highest exposure group with the lowest exposure group. We will then compare the incremental risk increase from one unit of exposure to a lower unit of exposure. Where authors of studies have reported their exposure in exposure categories, we will follow the aforementioned procedure proposed by (Il’yasova et al., 2005).

If a meta-analytical approach is not viable we will consider synthesis using other methods such as summarising effect estimates, combining p-values or vote counting based on direction of effects (McKenzie and Brennan, 2019). We will utilise the Synthesis Without Meta-analysis reporting guidelines to record our narrative synthesis and approach transparently (Campbell et al., 2020).

#### 3.6.1 Assessment of heterogeneity

We will quantify the statistical heterogeneity between studies by the tau2 statistic and calculate the 95% prediction interval based on the tau2 measure. We will consider statistical heterogeneity as considerable if the prediction interval includes 1 as a measure of no effect.

For clinical heterogeneity we will consider studies including men separately to those including women. Additionally, we will consider the general population separately from occupational studies. We will consider RF-EMF from all sources as similar. As previously mentioned, it is likely that studies will present whole-body RF-EMF exposure, and this may be from multiple sources (e.g., mobile phones, WLAN, base stations). Where possible we will conduct sub-group analyses for different sources of RF-EMF. We will consider the different outcomes separately.

### 3.7 Additional analyses

#### 3.7.1 Subgroup analyses

We will use sub-group analyses or meta-regression as appropriate to better understand which study-level factors may drive heterogeneity or modification of effect measures. For both male and female reviews subgroup analyses will be performed on the method of exposure measure, type of exposure (e.g. mobile phone, transmitter, etc), and whether the exposure was localised or non-localised. For occupational studies a subgroup analysis will be performed if feasible based on job. Meta-regression will be conducted only if ten or more studies are included in the meta-analysis (Deeks et al., 2021) on the following subgroups: study design (cohort versus case-control), risk of bias (high versus low), type of analysis (e.g. different adjustment factors), date of publication, geographical setting, and sample size. Each study will be weighted in the regression models using the inverse of its variance; studies with the lowest variance will be assigned greater weight in the regression model than those with the largest variance. We will show the association between each exposure and outcome of interest in table format where, for each variable, we will report its regression coefficient, standard error, 95% CI and statistical significance.

Variables that will be considered as sources of heterogeneity are date of publication; study design; geographical setting; sample size; method of exposure measure; and risk of bias (e.g. low vs high)

As previously mentioned, the variables that will be considered as confounders are mean age; sex; SES (known to be associated with increased environmental exposures, as this can result in less agency in determining where one lives and works); ethnicity; co-exposures (e.g. hazardous materials and heat) and the effect of dosage of RF-EMF within the pre-determined exposure categories. Any further confounders highlighted during data extraction will be carefully considered.

For clinical heterogeneity we will consider studies including men separately to those including women. Additionally, we will consider studies assessing the general population separately from those assessing occupational samples.

#### 3.7.2 Sensitivity analyses

Sensitivity analysis will be used to assess the impact of any assumptions that we make in the review process for example we will conduct sensitivity analysis of assumptions we make to allow assessment of exposure categories to be made; we will also complete a leave one out meta-analysis to identify whether an individual study is unduly affecting estimates.

The effect of the risk of bias will be examined by comparing the results of the overall analyses versus the results of only studies that are at low risk of bias.

### 3.8 Certainty of evidence assessment

The OHAT GRADE approach for observational studies will be used to qualify the certainty of the evidence per outcome for each category of exposure. We will not utilise the extra domain for coherence of evidence streams as suggested by OHAT because we will consider only one evidence stream in this review. Two reviewers will separately undertake the assessment and any disagreement will be resolved using moderation by a third reviewer if needed.

## Funding

All authors are salaried staff members of their respective institutions. The publication was prepared with financial support from the 10.13039/100004423World Health Organization.

## Author contributions

FP identified and coordinated the collaborative review team and topic experts including RPWK, EBM, CR, FB, DC, AA, JR, MP, MT and CC. MP is an expert in epidemiology and ionising radiation exposure assessment; AA, JR and MT are experts in perinatal epidemiology; CC and MT are experts in EMF-RF exposure assessment; and RPWK, EBM, FP, DC are experts in systematic review methods. CR and FB are information Specialists.

FP led and RPWK, EBM, CR, AA, JR, CC and MT contributed to the development and writing of the protocol. The search strategy was developed and piloted by CR and FB in collaboration with CC, EBM, AA, FP, and JR.

All authors made substantial contributions to the revisions of the manuscript.

## CRediT authorship contribution statement

**Ryan P.W. Kenny, Evelyn Barron Millar, Adenike Adesanya, Catherine Richmond, Carolina Calderon, Judith Rankin, Mireille Toledano, Maria Feychting :** Methodology, Writing - original draft, review & editing. **Fiona Pearson:** Funding acquisition, Project administration, Methodology, Supervision, Writing - original draft, review & editing. **Dawn Craig:** Methodology, Supervision, Writing - original draft. **Mark S Pearce:** Methodology, Writing - original draft. **Fiona Beyer:** Project administration, Supervision, Writing - original draft.

## Declaration of Competing Interest

The authors declare the following financial interests/personal relationships which may be considered as potential competing interests: Maria Feychting has a permanent position as Professor of Epidemiology at Karolinska Institutet, Stockholm Sweden since 2005. She has served as advisor to a number of national and international public advisory and research steering groups concerning the potential health effects of exposure to non-ionizing radiation, including the WHO (ongoing), Public Health England Advisory Group on Non-ionising Radiation - AGNIR (2009–2017), the Norwegian Public Health Institute (2010–2012), the Swedish Council for Working Life and Social Research (2003–2012), the Swedish Radiation Safety Authority’s independent scientific expert group on electromagnetic fields (2003–2011). She was member of the International Commission on Non-Ionizing Radiation Protection (ICNIRP), an independent body setting guidelines for non-ionizing radiation protection (2008–May 2020), and vice chairman of the Commission (May 2016–May 2020).

Mireille Toledano has been involved in funded research assessing mobile phone and other wireless technologies usage on health outcomes. The SCAMP (study cognition adolescents and mobile phones) prospective cohort study which is currently ongoing (2015–2021). The COSMOS (cohort study of mobile phone use and health) a longitudinal cohort study which is completed (2019).
