# Supplementary material for: The effects of radiofrequency exposure on male fertility and adverse reproductive outcomes: A protocol for two systematic reviews of human observational studies with meta-analysis
Source: Environ Int. 2022 Jan;158:106968. doi: 10.1016/j.envint.2021.106968 (PMC8669072; doi:10.1016/j.envint.2021.106968)
Supplement: Supplementary Data 2 [file mmc2.pdf]

Supplementary File 2

Search strategies for each database

Medline search v1.0 – Run on 19th November 2020

Database(s): **Ovid MEDLINE(R) and Epub Ahead of Print, In-Process & Other Non-Indexed Citations, Daily and Versions(R)** 1946 to November 17, 2020
Search Strategy:

#	Searches	Results
1	"Cell Phone Use"/	221
2	exp Cell Phone/	11043
3	(smartphone or cellphone or iphone).ti,ab,kw.	11256
4	((cell* or mobile or car or smart or dect or cordless) adj (phone* or telephone* or device* or handset* or communication*)).ti,ab,kw.	26698
5	exp Radio Waves/	23796
6	Radio/	2191
7	("radio wave*" or radiowave* or "micro wave*" or microwave*).ti,ab,kw.	38690
8	(("non ioni#ing" or nonioni#ing) adj radiation).ti,ab,kw.	902
9	Electromagnetic Fields/	17191
10	((radiofrequency or electromagnetic) adj2 (field* or exposure* or radiation or wave* or energy)).ti,ab,kw.	19955
11	((radio or broadcast*) adj transmitter*).ti,ab,kw.	326
12	broadcasting.ti,ab,kw.	1059
13	((radio or telegraph) adj operator*).ti,ab,kw.	62
14	Radar/	1230
15	radar*.ti,ab,kw.	5536
16	"base station*".ti,ab,kw.	926
17	(mast adj3 (phone* or telephone* or mobile* or network* or communication* or television* or TV or radio)).ti,ab,kw.	76
18	"plastic sealer*".ti,ab,kw.	9
19	(FM adj (radio or signal*)).ti,ab,kw.	166
20	Television/	13503

21	((television or TV) adj signal).ti,ab,kw.	23
22	((1G or 2G or 3G or 4G or 5G) and network*).ti,ab,kw.	458
23	"access point*".ti,ab,kw.	932
24	"Total Access Communication System".ti,ab,kw.	2
25	(TACS and (phone* or telephone* or communication* or network*)).ti,ab,kw.	137
26	"Code-division multiple access".ti,ab,kw.	212
27	(CDMA or WCDMA).ti,ab,kw.	223
28	"Global System for Mobile*".ti,ab,kw.	325
29	(GSM and (phone* or telephone* or communication* or network*)).ti,ab,kw.	703
30	"digital cellular system".ti,ab,kw.	10
31	(dcs and (phone* or telephone* or communication* or network*)).ti,ab,kw.	720
32	"Universal Mobile Telecommunication*".ti,ab,kw.	89
33	(UMTS and (phone* or telephone* or communication* or network*)).ti,ab,kw.	134
34	"Long-Term Evolution".ti,ab,kw.	1515
35	(LTE and (phone* or telephone* or communication* or network*)).ti,ab,kw.	135
36	"Worldwide Interoperability for Microwave Access".ti,ab,kw.	17
37	WiMax.ti,ab,kw.	72
38	"Terrestrial Trunked Radio".ti,ab,kw.	22
39	(Tetra and radio).ti,ab,kw.	48
40	"Digital Enhanced Cordless Telecommunication*".ti,ab,kw.	19
41	Wireless Technology/	3667
42	((wifi or "wi fi" or wireless or broadband or bluetooth or "blue tooth" or laptop*) and (signal* or network* or communication* or frequenc* or output*)).ti,ab,kw.	17952
43	(WLAN or "Wireless Local Area Network").ti,ab,kw.	249
44	((millimetre or millimeter or mm) adj wave*).ti,ab,kw.	1517
45	Satellite Communications/	1238

46	"satellite communication*".ti,ab,kw.	166
47	(uplink or downlink).ti,ab,kw.	407
48	exp Telemetry/	13525
49	telemetry.ti,ab,kw.	7217
50	("w/kg" or "specific absorption rate").ti,ab,kw.	4427
51	or/1-50	174390
52	Radiofrequency Ablation/	949
53	51 not 52	174248
54	exp Infertility, Male/	28090
55	(aspermia or asthenozoospermia or azoospermia or oligospermia or "sertoli cell-only syndrome" or teratozoospermia).ti,ab,kw.	8745
56	(sperm adj1 (count or quality or motility or abnormal or mobility or morphology)).ti,ab,kw.	19341
57	((infertil* or fertil*) adj1 (male* or man or men)).ti,ab,kw.	19712
58	or/54-57	52558
59	51 and 58	266
60	Infertility, Female/	28666
61	((infertil* or fertil*) adj1 (female* or wom#n)).ti,ab,kw.	7200
62	exp Abortion, Spontaneous/	35413
63	Aborted Fetus/	1229
64	exp Abortion, Induced/	40355
65	miscarr*.ti,ab,kw.	14890
66	(abort* adj1 (spontaneous or missed or incomplete or habitual or induce* or therapeutic or eugenic)).ti,ab,kw.	20338
67	Fetal Growth Retardation/	16481
68	((f?etal or intrauterine) adj "growth retardation").ti,ab,kw.	6984
69	Stillbirth/	4973
70	(stillbirth or stillborn or "still born").ti,ab,kw.	11749

71	exp Infant, Low Birth Weight/	34758
72	Sex Ratio/	9304
73	"sex ratio*".ti,ab,kw.	15141
74	exp twinning, embryonic/	234
75	exp Multiple Birth Offspring/	28545
76	("multiple birth*" or twins or triplets or quadruplets or quintuplets or twinning).ti,ab,kw.	39650
77	((congenital or developmental or embryonic) adj1 (disorder* or disease* or condition* or abnormalit* or malformation* or anomal* or defect*)).ti,ab,kw.	75853
78	exp Congenital Abnormalities/	596603
79	Maternal Exposure/	9528
80	Prenatal Exposure Delayed Effects/	29745
81	"prenatal development*".ti,ab,kw.	2270
82	exp "Embryonic and Fetal Development"/	277801
83	((embryonic or f?etal) adj (development or death)).ti,ab,kw.	46769
84	exp Pregnancy Trimesters/ or exp Pregnancy/ or exp Pregnancy Outcome/ or exp Pregnancy Complications/	931381
85	exp Pregnant Women/	8626
86	exp Parturition/	17522
87	exp Gravidity/	1201
88	exp Parity/	25185
89	exp Infant, Newborn/	613337
90	exp Fetus/	157975
91	pregnan*.ti,ab,kw.	531613
92	(prenatal or "pre natal" or antenatal or "ante natal" or antepartum or "ante partum" or postnatal or "post natal" or postpartum or "post partum" or perinatal or "peri natal" or peripartum or "peri partum").ti,ab,kw.	334189
93	(matern* or mother* or baby or babies or f?etal or f?etus or neonat* or newborn or "new born").ti,ab,kw.	939198
94	(birth* or childbirth).ti,ab,kw.	355245

95	(preterm or "pre term").ti,ab,kw.	77034
96	gestation*.ti,ab,kw.	212626
97	or/60-96	2716896
98	51 and 97	7253
99	59 or 98	7481

Embase v1.0 - run on 19th November 2020

Database(s): **Embase** 1974 to 2020 November 18

Search Strategy:

#	Searches	Results
1	"Cell Phone Use"/	921
2	exp mobile phone/	30843
3	(smartphone or cellphone or iphone).ti,ab,kw.	15549
4	((cell* or mobile or car or smart or dect or cordless) adj (phone* or telephone* or device* or handset* or communication*)).ti,ab,kw.	36359
5	radiofrequency radiation/	6255
6	Radio/	630
7	("radio wave*" or radiowave* or "micro wave*" or microwave*).ti,ab,kw.	43457
8	(("non ioni#ing" or nonioni#ing) adj radiation).ti,ab,kw.	1234
9	electromagnetism/	4467
10	((radiofrequency or electromagnetic) adj2 (field* or exposure* or radiation or wave* or energy)).ti,ab,kw.	21228
11	((radio or broadcast*) adj transmitter*).ti,ab,kw.	355
12	broadcasting.ti,ab,kw.	1142
13	((radio or telegraph) adj operator*).ti,ab,kw.	57
14	telecommunication/	25232
15	radar*.ti,ab,kw.	5320
16	"base station*".ti,ab,kw.	1044
17	(mast adj3 (phone* or telephone* or mobile* or network* or communication* or television* or TV or radio)).ti,ab,kw.	104
18	"plastic sealer*".ti,ab,kw.	11
19	(FM adj (radio or signal*)).ti,ab,kw.	171
20	Television/	13948
21	((television or TV) adj signal).ti,ab,kw.	25
22	((1G or 2G or 3G or 4G or 5G) and network*).ti,ab,kw.	621

23	"access point*".ti,ab,kw.	1303
24	"Total Access Communication System".ti,ab,kw.	3
25	(TACS and (phone* or telephone* or communication* or network*)).ti,ab,kw.	246
26	"Code-division multiple access".ti,ab,kw.	157
27	(CDMA or WCDMA).ti,ab,kw.	228
28	"Global System for Mobile*".ti,ab,kw.	372
29	(GSM and (phone* or telephone* or communication* or network*)).ti,ab,kw.	902
30	"digital cellular system".ti,ab,kw.	9
31	(dcs and (phone* or telephone* or communication* or network*)).ti,ab,kw.	1074
32	"Universal Mobile Telecommunication*".ti,ab,kw.	90
33	(UMTS and (phone* or telephone* or communication* or network*)).ti,ab,kw.	163
34	"Long-Term Evolution".ti,ab,kw.	2095
35	(LTE and (phone* or telephone* or communication* or network*)).ti,ab,kw.	155
36	"Worldwide Interoperability for Microwave Access".ti,ab,kw.	16
37	WiMax.ti,ab,kw.	78
38	"Terrestrial Trunked Radio".ti,ab,kw.	23
39	(Tetra and radio).ti,ab,kw.	74
40	"Digital Enhanced Cordless Telecommunication*".ti,ab,kw.	20
41	wireless communication/	5523
42	((wifi or "wi fi" or wireless or broadband or bluetooth or "blue tooth" or laptop*) and (signal* or network* or communication* or frequenc* or output*)).ti,ab,kw.	18613
43	(WLAN or "Wireless Local Area Network").ti,ab,kw.	277
44	((millimetre or millimeter or mm) adj wave*).ti,ab,kw.	987
45	"satellite communication*".ti,ab,kw.	166
46	(uplink or downlink).ti,ab,kw.	316
47	exp Telemetry/	29542

48	telemetry.ti,ab,kw.	11715
49	("w/kg" or "specific absorption rate").ti,ab,kw.	4962
50	or/1-49	217724
51	exp male infertility/	42437
52	(aspermia or asthenozoospermia or azoospermia or oligospermia or "sertoli cell-only syndrome" or teratozoospermia).ti,ab,kw.	12399
53	(sperm adj1 (count or quality or motility or abnormal or mobility or morphology)).ti,ab,kw.	25827
54	((infertil* or fertil*) adj1 (male* or man or men)).ti,ab,kw.	27711
55	or/51-54	70391
56	50 and 55	435
57	exp female infertility/	45057
58	((infertil* or fertil*) adj1 (female* or wom#n)).ti,ab,kw.	17294
59	spontaneous abortion/	40601
60	exp fetus death/	38389
61	exp induced abortion/	29184
62	miscarr*.ti,ab,kw.	25476
63	(abort* adj1 (spontaneous or missed or incomplete or habitual or induce* or therapeutic or eugenic)).ti,ab,kw.	24278
64	exp intrauterine growth retardation/	45284
65	((f?etal or intrauterine) adj "growth retardation").ti,ab,kw.	9560
66	Stillbirth/	17906
67	(stillbirth or stillborn or "still birth").ti,ab,kw.	16486
68	exp low birth weight/	63530
69	Sex Ratio/	71846
70	"sex ratio*".ti,ab,kw.	20418
71	exp twinning/	147
72	exp Multiple Birth Offspring/	39510

73	("multiple birth*" or twins or triplets or quadrulets or quintuplets or twinning).ti,ab,kw.	46852
74	((congenital or developmental or embryonic) adj1 (disorder* or disease* or condition* or abnormalit* or malformation* or anomal* or defect*)).ti,ab,kw.	103469
75	exp congenital disorder/	1346462
76	Maternal Exposure/	2684
77	prenatal exposure/	25180
78	"prenatal development*".ti,ab,kw.	2670
79	exp prenatal development/	225673
80	((embryonic or f?etal) adj (development or death)).ti,ab,kw.	58887
81	exp pregnancy/ or pregnancy outcome/ or exp pregnancy complication/	749798
82	pregnant woman/	83032
83	birth/	18131
84	parity/	36641
85	newborn/	536578
86	fetus/	194544
87	pregnan*.ti,ab,kw.	659092
88	(prenatal or "pre natal" or antenatal or "ante natal" or antepartum or "ante partum" or postnatal or "post natal" or postpartum or "post partum" or perinatal or "peri natal" or peripartum or "peri partum").ti,ab,kw.	437385
89	(matern* or mother* or baby or babies or f?etal or f?etus or neonat* or newborn or "new born").ti,ab,kw.	1158193
90	(birth* or childbirth).ti,ab,kw.	454801
91	(preterm or "pre term").ti,ab,kw.	110452
92	gestation*.ti,ab,kw.	295253
93	or/57-92	3507803
94	50 and 93	11652
95	56 or 94	12004

Medline search v0.6 translation to EMF database

searches run and exported on 17th and 18th November 2020. Not possible to build a strategy, so searched line by line and exported immediately. EMF portal added the non-English words. Frequency ranges included: radio frequency, mobile communications and low frequency. All topics and complete time span included.

Search Strategy:

#	Searches	EMF terms used	No of results
54	exp Infertility, Male/		
55	(aspermia or asthenozoospermia or azoospermia or oligospermia or "sertoli cell-only syndrome" or teratozoospermia).ti,ab,kw.	aspermia, asthenozoospermia, azoospermia, oligospermia, teratozoospermia<br>“sertoli cell-only syndrome”	7<br>0
56	(sperm adj1 (count or quality or motility or abnormal or mobility or morphology)).ti,ab,kw.	"sperm count", "sperm quality", "sperm motility", Spermienbeweglichkeit, Spermienmotilität, 精子運動能, "abnormal sperm", "sperm mobility", "sperm morphology"	166
57	((infertil* or fertil*) adj1 (male* or man or men)).ti,ab,kw.	infertility, Unfruchtbarkeit, Infertilität, 不妊, fertility, Fortpflanzungsfähigkeit, Fruchtbarkeit, Fertilität, 受胎力 (adjacency operator not available, so not restricted to male only)<br>fertile, infertile, Unfruchtbarkeit, Infertilität, infertility, 不妊	408<br>140

58	or/54-57		
59	51 and 58		
60	Infertility, Female/		
61	((infertil* or feril*) adj1 (female* or wom#n)).ti,ab,kw.	See line 57	
62	exp Abortion, Spontaneous/		
63	Aborted Fetus/		
64	exp Abortion, Induced/		
65	(miscarriage or miscarry).ti,ab,kw.	miscarriage, Fehlgeburt, Abort, abortion, 流産, Fehlgeburt, Abort, miscarriage, abortion, 流産, miscarry, miscarried	141
66	(abort* adj1 (spontaneous or missed or incomplete or habitual or induce* or therapeutic or eugenic)).ti,ab,kw.	Abort, Fehlgeburt, miscarriage, abortion, 流産, aborts, abortion, Fehlgeburt, Abort, miscarriage, 流産, Fehlgeburt, Abort, miscarriage, abortion, 流産, aborted	141
67	Fetal Growth Retardation/		
68	((f?etal or intrauterine) adj "growth retardation").ti,ab,kw.	"fetal growth retardation", "foetal growth retardation", "intrauterine growth retardation"	12
69	Stillbirth/		

70	(stillbirth or stillborn).ti,ab,kw.	"still born", stillborn, "still birth", stillbirth, Totgeburt, 死産	405
71	exp Infant, Low Birth Weight/		
72	("low birth weight" or "small for gestational age").ti,ab,kw.	"low birth weight", "small for gestational age"	32
73	Sex Ratio/		
74	"sex ratio*".ti,ab,kw.	"sex ratio", Geschlechtsverhältnis, 性比	56
75	exp twinning, embryonic/		
76	exp Multiple Birth Offspring/		
77	"multiple birth*".ti,ab,kw.	twins, triplets, quadruplets, quintuplets, twinning, "multiple birth"	75
78	((congenital or developmental or f?etal or prenatal or embryonic) adj1 (disorder* or disease* or condition* or abnormalit* or malformation* or anomal* or defect*)).ti,ab,kw.	Congenital<br>developmental<br>Other terms are included below	104<br>235
79	exp Congenital Abnormalities/		
80	Maternal Exposure/		
81	((prenatal or maternal) adj exposure*).ti,ab,kw.	Prenatal and maternal included below	
82	Prenatal Exposure Delayed Effects/		
83	"prenatal development*".ti,ab,kw.	Prenatal included below	

84	exp "Embryonic and Fetal Development"/		
85	((embryonic or f?etal) adj (development or death)).ti,ab,kw.	Embryo, embryonic<br><br>Fetal etc covered below	769
86	or/60-85		
87	51 and 86		
88	exp Pregnancy Trimesters/ or exp Pregnancy/ or exp Pregnancy Outcome/ or exp Pregnancy Complications/		
89	exp Pregnant Women/		
90	exp Parturition/		
91	exp Gravidity/		
92	exp Parity/		
93	exp Infant, Newborn/		
94	exp Fetus/		
95	pregnan*.ti,ab,kw.	Pregnant, pregnancies, Schwangerschaft, pregnancy, pregnancy, Schwangerschaft, Schwangerschaft, pregnancy	837
96	(prenatal or "pre natal" or antenatal or "ante natal" or antepartum or "ante partum" or postnatal or "post natal" or postpartum or "post partum" or perinatal or "peri natal" or peripartum or "peri partum").ti,ab,kw.	prenatal "pre natal" antenatal "ante natal" antepartum "ante partum prenatal, pränatal, 出生前の, "pre natal", antenatal, "ante natal", antepartum, "ante partum"	538

		postnatal "post natal" postpartum "post partum" perinatal "peri natal" peripartum "peri partum"	244
97	(matern* or mother* or baby or babies or f?etal or f?etus or neonat* or newborn or "new born").ti,ab,kw.	baby, babies, mother, mothers<br>maternal, mütterlich, 母親の, maternity<br>fetal, fetus, foetal, foetus, Fetus, Fötus, 胎児, Fetus, Fötus, 胎児, Fetus, Fötus, 胎児, Fetus, Fötus, 胎児<br>neonate, neonates, neonatal, neugeboren, newborn, 新生児の, 新生児<br>newborn, newborns “new born” neugeboren, neonatal, 新生児の, 新生児, neugeboren, neonatal, newborn, 新生児の, 新生児, neugeboren, neonatal, newborn, 新生児の, 新生児	240<br>262<br>751<br>468<br>463
98	(birth* or childbirth).ti,ab,kw.	Birth, births, birthed, birthing, childbirth, childbirths,	362
99	(preterm or "pre term").ti,ab,kw.	Preterm, “pre term”	33
100	gestation*.ti,ab,kw.	Gestation, gestations, gestational, Tragzeit, 妊娠	525
101	or/88-100		
102	51 and 101		
103	59 or 87		

104	102 or 103		
**Total number of records retrieved:**			**7414**
**Deduplicated total: (matched author, year, title, journal title)**			**2398**
