# Supplementary material for: The effects of radiofrequency exposure on male fertility and adverse reproductive outcomes: A protocol for two systematic reviews of human observational studies with meta-analysis
Source: Environ Int. 2022 Jan;158:106968. doi: 10.1016/j.envint.2021.106968 (PMC8669072; doi:10.1016/j.envint.2021.106968)
Supplement: Supplementary Data 4 [file mmc4.docx]

**Risk of bias assessment**

1. **Introduction**

The OHAT risk of bias tool for human and animal studies (NTP-OHAT, 2015) will be utilised for assessment of a study’s internal validity. All reviewers who undertake the risk of bias assessment will complete this in the excel data extraction sheet. For each question the reviewer will utilise the details below to consider each domain and provide an appropriate rating. These ratings will be given with supporting information identified in a cell next to the decision. Where appropriate reviewers will indicate the original source and page numbers.

One reviewer will complete the data extraction and risk of bias assessment, which will then be checked for consistency by a second reviewer. Conflicts will be resolved by discussion, if the two reviewers are unable to agree a third reviewer will arbitrate.

1. **Direction of bias**

Direction and magnitude of bias will be considered. A column is provided for each question with a dropdown menu of “upward, downward, or unpredictable” available. The classification of the bias direction will depend on the type of bias. For some questions or domains, the bias is most easily thought of as directional towards or away from the null, and for others (confounding, selection bias, and forms of measurement bias such as differential misclassification), the bias is thought of as an increase or decrease in the effect estimate independent of the null (NTP-OHAT, 2019). When the bias rating is (-) probably high or (--) definitely high, we will indicate whether the bias direction is:

- *Upward (e.g. toward the null),* resulting in apparent or overestimated effects of the exposure;
- *Downward (e.g. away from the null)*, resulting in undetected or underestimated effects of the exposure;
- *Unpredictable,* due to lack of ad hoc bias studies or generalizable external evidence.

However, as outlined by OHAT and COSTER guidelines the reviewer will not attempt to guess the direction of bias in the absence of a clear rationale with scientific support (NTP-OHAT, 2019; Whaley et al., 2020).

1. **Domains**

The below table provides an overview of each domain, and the study types that these questions will be answered for.

**Table 1.** Overview of risk of bias

| **Domain** | **Question** | **Applicable study designs** |
| --- | --- | --- |
| Selection bias | Did selection of study participants result in appropriate comparison groups? | Cohort  Case-control  Cross-sectional  Case series/Case report |
| Confounding bias | Did the study design or analysis account for important confounding and modifying variables? | Cohort  Case-control  Cross-sectional  Case series/Case report |
| Attrition bias | Were outcome data complete without attrition or exclusion from analysis? | Cohort  Case-control  Cross-sectional  Case series/Case report |
| Detection bias | Can we be confident in the exposure characterization? | Cohort  Case-control  Cross-sectional  Case series/Case report |
|  | Can we be confident in the outcome assessment? |  |
| Selective reporting bias | Were all measured outcomes reported? | Cohort  Case-control  Cross-sectional  Case series/Case report |
| Other bias | Were statistical methods appropriate? | Cohort  Case-control  Cross-sectional  Case series/Case report |

- 1. *Selection bias*

This section will consider whether there were any systematic differences between the baseline characteristics of the groups that are compared.

1. *Did selection of study participants result in appropriate comparison groups?*

Addresses whether exposed and unexposed subjects were recruited from the same populations in cohort or cross-sectional studies, and consideration of appropriate selection of cases and controls in case-control studies.

| Definitely Low Risk of Bias (++) |
| --- |
| - There is direct evidence that exposed and non-exposed subjects (or subgroups by exposure level) were recruited from the same eligible population within the same time frame, with the same method of ascertainment, using the same inclusion and exclusion criteria, - **AND** probability of enrolment/participation did not depend on *both* exposure status and risk profile for the outcome(s) of interest. |
| - **Probably Low Risk of Bias (+)** |
| - There is indirect evidence that exposed and non-exposed subjects (or subgroups by exposure level) were recruited from the same eligible population within the same time frame, with the same method of ascertainment, using the same inclusion and exclusion criteria, - **AND** probability of enrolment/participation did not depend on *both* exposure status and risk profile for the outcome(s) of interest, - **OR** differences between groups would not appreciably bias results. |
| - **Probably High Risk of Bias (-) or (NR)** |
| - There is indirect evidence that exposed and non-exposed subjects (or subgroups by exposure level) were not recruited from the same eligible population, or were recruited within very different time frames, - **AND** probability of enrolment/participation depended on both exposure status and risk profile for the outcome of interest (selection bias), - **OR** there is insufficient information about the unexposed/comparison group, including a different rate of non-response without an explanation (record “NR” as basis for answer). |
| - **Definitely High Risk of Bias (--)** |
| - There is direct evidence that exposed and non-exposed subjects (or subgroups by exposure level) were not recruited from the same eligible population, or were recruited within very different time frames, or had very different participation/response rates, - **AND** probability of enrolment/participation depended on both exposure status and risk profile for the outcome(s) of interest (selection bias). |

*3.2 Confounding bias*

*4. Did the study design or analysis account for important confounding and modifying variables?*

Confounding variables include any factor that is: 1) associated with exposure, 2) an independent risk factor for a given outcome, and 3) unequally distributed between study groups (Gerstman, 2013). Appropriate methods to account for these differences would include multivariable analysis, stratification, matching of cases and controls, or other similar approaches. Consider how the statistical analysis was completed and whether appropriate confounding factors were included. The following critical confounder relationships have been identified by experts in the RF-EMF field and will be assessed for both SR3A and SR3B: age, ethnicity, BMI, socioeconomic status (SES), smoking status, alcohol intake. The following confounders are of importance but not critical: geographical location, co-exposures (e.g. occupation exposure to hazardous substances and heat), environmental noise and air pollution. Confounders that may only effect pregnant women include maternal or gestational morbidities, folic acid intake, new-born gender, consanguinity, place of birth (setting), and whether the pregnancy is a multiple. Arbitrarily a minimum of three critical confounding factors should be accounted for in order that a study be deemed at low risk of bias. If a study’s analysis includes less than three critical confounders, consider the importance of the other factors included. In this instance, a definitely low risk of bias is not plausible. However, a low risk of bias may still be achieved if other important factors are assessed. Studies that do not include any confounders will be rated as at high risk of bias.

| Definitely Low Risk of Bias (++) |
| --- |
| - There is direct evidence that appropriate adjustments or explicit considerations were made for primary covariates and confounders in the final analyses through the use of statistical models to reduce research-specific bias including standardization, matching, adjustment in multivariate model, stratification, propensity scoring, or other methods that were appropriately justified. Acceptable consideration of appropriate adjustment factors includes cases when the factor is not included in the final adjustment model because the author conducted analyses that indicated it did not need to be included, - **AND** there is direct evidence that primary covariates and confounders were assessed using valid and reliable measurements, - **AND** there is direct evidence that other exposures anticipated to bias results were not present or were appropriately measured and adjusted for. In occupational studies or studies of contaminated sites, other chemical exposures known to be associated with those settings were appropriately considered. |
| - **Probably Low Risk of Bias (+)** |
| - There is indirect evidence that appropriate adjustments were made, - **OR** it is deemed that not considering or only considering a partial list of covariates or confounders in the final analyses would not appreciably bias results. - **AND** there is evidence (direct or indirect) that primary covariates and confounders were assessed using valid and reliable measurements, - **OR** it is deemed that the measures used would not appreciably bias results (i.e., the authors justified the validity of the measures from previously published research), - **AND** there is evidence (direct or indirect) that other co-exposures anticipated to bias results were not present or were appropriately adjusted for, - **OR** it is deemed that co-exposures present would not appreciably bias results. |
| - **Probably High Risk of Bias (-) or (NR)** |
| - There is indirect evidence that the distribution of primary covariates and known confounders differed between the groups (or cases and controls, that was not investigated further) and was not appropriately adjusted for in the final analyses, - **OR** there is insufficient information provided about the distribution of known confounders (record “NR” as basis for answer), - **OR** there is indirect evidence that primary covariates and confounders were assessed using measurements of unknown validity, - **OR** there is insufficient information provided about the measurement techniques used to assess primary covariates and confounders (record “NR” as basis for answer), - **OR** there is indirect evidence that there was an unbalanced provision of additional co-exposures across the primary study groups, which were not appropriately adjusted for, - **OR** there is insufficient information provided about co-exposures in occupational studies or studies of contaminated sites where high exposures to other chemical exposures would have been reasonably anticipated (record “NR” as basis for answer). |
| - **Definitely High Risk of Bias (--)** |
| - There is direct evidence that the distribution of primary covariates and known confounders differed between the groups (or cases and controls), confounding was demonstrated, and was not appropriately adjusted for in the final analyses, - **OR** there is direct evidence that primary covariates and confounders were assessed using non valid measurements, - **OR** there is direct evidence that there was an unbalanced provision of additional co-exposures across the primary study groups, which were not appropriately adjusted for. |

- 1. *Attrition/exclusion bias*

*7. Were outcome data complete without attrition or exclusion from analysis?*

Consider the outcome data and if there were any dropouts, nonresponses, loss to follow-up or exclusions from the analysis.

| Definitely Low Risk of Bias (++) |
| --- |
| - There is direct evidence that there was no loss of subjects during the study and outcome data were complete, - **OR** loss of subjects (i.e., incomplete outcome data) was adequately addressed and reasons were documented when human subjects were removed from a study or analyses. Review authors should be confident that the participants included in the analysis are exactly those who were randomized into the trial. Acceptable handling of subject attrition includes: very little missing outcome data (less than 20% in each group (Genaidy et al. 2007)); reasons for missing subjects unlikely to be related to outcome; missing outcome data balanced in numbers across study groups, with similar reasons for missing data across groups, - **OR** analyses (such as intention-to-treat analysis) in which missing data have been imputed using appropriate methods (insuring that the characteristics of subjects lost to follow up or with unavailable records are described in identical way and are not significantly different from those of the study participants). - There is direct evidence that loss of subjects (i.e., incomplete outcome data) was adequately addressed and reasons were documented when human subjects were removed from a study. Acceptable handling of subject attrition includes: very little missing outcome data; reasons for missing subjects unlikely to be related to outcome (for survival data, censoring unlikely to be introducing bias); missing outcome data balanced in numbers across study groups, with similar reasons for missing data across groups, - **OR** missing data have been imputed using appropriate methods and characteristics of subjects lost to follow up or with unavailable records are described in identical way and are not significantly different from those of the study participants. - There is direct evidence that exclusion of subjects from analyses was adequately addressed, and reasons were documented when subjects were removed from the study or excluded from analyses. - **Note:** Participants randomized but subsequently found not to be eligible need not always be considered as having missing outcome data (Higgins and Green 2011). |
| Probably Low Risk of Bias (+) |
| - There is indirect evidence that loss of subjects (i.e., incomplete outcome data) was adequately addressed and reasons were documented when human subjects were removed from a study, - **OR** it is deemed that the proportion lost to follow-up would not appreciably bias results (less than 20% in each group (Genaidy et al. 2007)). This would include reports of no statistical differences in characteristics of subjects lost to follow up or with unavailable records from those of the study participants. Generally, the higher the ratio of participants with missing data to participants with events, the greater potential there is for bias. For studies with a long duration of follow-up, some withdrawals for such reasons are inevitable. - There is indirect evidence that loss of subjects (i.e., incomplete outcome data) was adequately addressed and reasons were documented when human subjects were removed from a study, - **OR** it is deemed that the proportion lost to follow-up would not appreciably bias results. This would include reports of no statistical differences in characteristics of subjects lost to follow up or with unavailable records from those of the study participants. Generally, the higher the ratio of participants with missing data to participants with events, the greater potential there is for bias. For studies with a long duration of follow-up, some withdrawals for such reasons are inevitable. - There is indirect evidence that exclusion of subjects from analyses was adequately addressed, and reasons were documented when subjects were removed from the study or excluded from analyses. |
| Probably High Risk of Bias (-) |
| - There is indirect evidence that loss of subjects (i.e., incomplete outcome data) was unacceptably large (greater than 20% in each group (Genaidy et al. 2007)) and not adequately addressed, - **OR** there is insufficient information provided about numbers of subjects lost to follow-up (record “NR” as basis for answer). - There is indirect evidence that loss of subjects (i.e., incomplete outcome data) was unacceptably large and not adequately addressed, - **OR** there is insufficient information provided about numbers of subjects lost to follow-up (record “NR” as basis for answer). - There is indirect evidence that exclusion of subjects from analyses was not adequately addressed, - **OR** there is insufficient information provided about why subjects were removed from the study or excluded from analyses (record “NR” as basis for answer). |
| Definitely High Risk of Bias (--) |
| - There is direct evidence that loss of subjects (i.e., incomplete outcome data) was unacceptably large and not adequately addressed. Unacceptable handling of subject attrition includes: reason for missing outcome data likely to be related to true outcome, with either imbalance in numbers or reasons for missing data across study groups; or potentially inappropriate application of imputation. - **OR** There is direct evidence that exclusion of subjects from analyses was not adequately addressed. Unacceptable handling of subject exclusion from analyses includes: reason for exclusion likely to be related to true outcome, with either imbalance in numbers or reasons for exclusion across study groups. |

- 1. *Detection bias*

Consider bias regarding how the experimental and control groups outcomes and exposures were assessed. Especially consider the validity and reliability of the methods. For example, self-reported outcomes can be prone to recall bias in retrospective exposure assessments (Savitz & Wellenius, 2016; Catalogue of Bias Collaboration 2017). Cohort or case-control analyses of cohort studies with prospective exposure assessment are not affected by recall bias.

*8. Can we be confident in the exposure characterization?*

The potential for different types of exposure misclassification depends on whether the exposure is assessed prospectively (i.e., before and independently of the outcome) or retrospectively (after the outcome occurrence/diagnosis/ascertainment), which is a feature of the study design. Ideally, studies will be prospective in nature.

Recall bias is reduced in case-control studies when the data is collected via In-person interviews are preferred over phone interviews, as is information obtained from the participant compared to a proxy source (Hutter et al., 2012). Investigators/interviews can be blinded to exposure information, outcome information, and the actual hypothesis underlying the study that links the exposure and outcome information (Parker & Berman, 2016). Mailed questionnaires are particularly prone to recall bias from lack of blinding on health status, due to a lack of blinding to the outcome than the participants (Bowling, 2005). Consider question types asked for self-reported data, with open ended questions (continuous data) being preferred due to greater accuracy (Boase & Ling, 2013; Mireku et al., 2018). Additionally, consider the time variance of the exposure with assessments of changes in frequency/intensity over time being preferred (Pettersson et al., 2014).

It is most likely that studies will report the outcomes as whole-body exposure, rather than area specific (i.e. abdominal region). Whilst this is a limitation of the findings this will be considered during the GRADE process and should not be grounds for downgrading here. As we are concerned with the accuracy of the measurements used rather than directness.

Focus on the implications of the exposure assessment method on the validity of each exposure contrast of interest for the review and assess the potential for specific types of exposure misclassification (see the blue box at the bottom of the table below). For studies investigating the effect of more than one RF-emitting equipment or device, rate the potential for exposure-information bias separately for each type of exposure source. Please note that concern is based on generalizable information from other relevant exposure validation studies.

| Definitely Low Risk of Bias (++) |
| --- |
| - The exposure was assessed *prospectively*, *independent of the outcome*, and *consistently* across study groups, that is, over the same time frame and using the same method, consisting of measured electric or magnetic field levels or power density of RF signals exposed to. For occupational studies it should include the worker location during relevant tasks, supplemented by historical information (at the individual level) on proximity to the equipment during relevant tasks, and task frequency and duration, including changes over time in any of the parameters. - **AND** exposure was assessed in a time-window relevant for outcome development (e.g. prior to infertility issues). |
| Probably Low Risk of Bias (+) |
| - The exposure was assessed *prospectively* and *consistently* across groups, using the methods and metrics described above, but there was no information about job/task characteristics or other details, **with** evidence that lack of this information about not appreciably affect risk estimates, - **OR** exposure was assessed using *prospectively collected self-reported information* (e.g., through questionnaire/interview prior to disease occurrence) about jobs/tasks involving exposure to RF-EMF including task frequency and duration, **with** evidence that information about jobs/tasks/activities is representative for the exposure time window of interest, - **OR** estimates of cumulative exposure were obtained combining RF exposure levels from source- or job-exposure matrices (SEM or JEM) with *occupational histories from independent sources* (e.g., census data), or with *prospectively collected self-reported occupational histories* and jobs/tasks with or nearby RF-sources (e.g., through questionnaire/interview before disease occurrence), - **OR** exposure was assessed using *retrospectively collected self-reported information* (e.g., through questionnaire/interview after disease occurrence) about jobs/tasks involving exposure to RF-EMF including task frequency and duration, **validated** and convincingly showing that reporting was not affected by the outcome (no recall bias), - **OR** estimates of cumulative exposure were obtained combining RF exposure levels from SEM/JEM with *retrospectively collected self-reported* occupational histories and jobs/tasks with or nearby RF-sources (e.g., through questionnaire/interview after disease occurrence), **validated** and convincingly showing that that reporting was not affected by the outcome (no recall bias), - **AND** exposure was assessed in a relevant time-window for development of the outcome development (e.g. prior to infertility issues). |
| Probably High Risk of Bias (-) or (NR) |
| - The exposure was assessed through retrospectively collected self-reported information about jobs/tasks involving exposure to RF-EMF, including task frequency and duration, **with validation** showing that reporting was likely affected by the outcome (i.e., evidence of recall bias), - **OR** estimates of cumulative exposure were obtained combining RF exposure levels from SEM/JEM with retrospectively collected self-reported occupational histories and jobs/tasks with or nearby RF-sources (e.g., through questionnaire/interview after disease occurrence), **with validation** showing that the exposure variable was likely affected by the outcome (i.e., evidence of recall bias), - **OR** there is insufficient information provided about the exposure assessment, including validity and reliability (record “NR” as basis for answer). |
| Definitely High Risk of Bias (--) |
| - The exposure was assessed using poorly validated methods (i.e., retrospective self-reported information about jobs/tasks involving exposure to RF-EMF, that does not capture any changes in exposure level over time), - **OR** there is evidence that exposure occurred outside the appropriate time window to potentially affect the outcome (e.g. prior to infertility issues), **OR** there is evidence for concern about the method used (Note 1). |
| **In case of (-) or (- -) rating, for each exposure contrast, specify whether concern is for** (toggle more than one if appropriate):   1. **Ever vs never exposed**  - Random exposure misclassification - Differential exposure misclassification (recall bias)  1. **Time since start exposure or exposure duration**  - Random exposure misclassification - Outcome-dependent exposure misclassification (reverse causation) - Differential exposure misclassification (recall bias)  1. **Average or cumulative exposure level**  - Random exposure misclassification - Misclassification dependent on true exposure (systematic error) - Misclassification dependent on outcome (reverse causation) - Differential misclassification (recall bias) |

*9. Can we be confident in the outcome assessment?*

Consider the outcome assessment applied, was it consistent (i.e. under the same method and time-frame).

| Definitely Low Risk of Bias (++) |
| --- |
| - There is direct evidence that the outcome was assessed using well-established methods (e.g., the “gold standard” with validity and reliability >0.70 (Genaidy et al. 2007)) that capture exposure measures (e.g. reference to a publication of the specific test/task is available and/or reference to a functionally equivalent is provided). - **AND** subjects had been followed for the same length of time in all study groups, using appropriate methods. - **AND** there is direct evidence that the outcome assessors (including study subjects, if outcomes were self-reported) were adequately blinded to the study group, and it is unlikely that they could have broken the blinding prior to reporting outcomes. - There is direct evidence that the outcome was assessed in cases (i.e., case definition) and controls using well-established methods (the gold standard), - **AND** subjects had been followed for the same length of time in all study groups, - **AND** there is direct evidence that the outcome assessors (including study subjects, if outcomes were self-reported) were adequately blinded to the exposure level when outcome was assessed in cases (i.e., case definition) and controls. |
| Probably Low Risk of Bias (+) |
| - There is indirect evidence that the outcome was assessed using acceptable methods (i.e., deemed valid and reliable but not the gold standard, with validity and reliability ≥0.40, Genaidy et al. 2007), that capture exposure, but no reference to a publication of the specific test/task/test battery is available and/or reference to a functionally equivalent is provided. - **AND** assessment methods are acceptable, - **AND** there is indirect evidence that the outcome assessors (including study subjects) were adequately blinded to the study group, and it is unlikely that they could have broken the blinding prior to reporting outcomes, - **OR** it is deemed that lack of adequate blinding of outcome assessors would not appreciably bias results, which is more likely to apply to objective outcome measures. |
| Probably High Risk of Bias (-) |
| - There is indirect evidence that the outcome assessment method is an insensitive instrument. - **AND** assessment methods are acceptable, - **OR** there is indirect evidence that the outcome assessors (including study subjects) were adequately blinded to the study group, and it is unlikely that they could have broken the blinding prior to reporting outcomes, - **OR** there is insufficient information provided about blinding of outcome assessors (record “NR” as basis for answer). |
| Definitely High Risk of Bias (--) |
| - There is direct evidence that the outcome assessment method is an insensitive instrument, and no information on test/task procedure and outcome parameter are reported. - **OR** there is direct evidence for lack of adequate blinding of outcome assessors (including study subjects if outcomes were self-reported), including no blinding or incomplete blinding. |

- 1. *Selective Reporting bias*

Selective reporting is present if pre-specified outcomes are not reported or incompletely reported.

*10. Were all measured outcomes reported?*

| Definitely Low Risk of Bias (++) |
| --- |
| - There is direct evidence that all of the study’s measured outcomes (primary and secondary) outlined in the protocol, methods, abstract, and/or introduction, or mentioned in the discussion (that are relevant for the evaluation) have been reported. This would include outcomes reported with sufficient detail to be included in meta-analysis or fully tabulated during data extraction and analyses had been planned in advance. |
| Probably Low Risk of Bias (+) |
| - There is indirect evidence that all of the study’s measured outcomes (primary and secondary) outlined in the protocol, methods, abstract, and/or introduction, or mentioned in the discussion (that are relevant for the evaluation) have been reported, - **OR** analyses that had not been planned in advance (i.e., retrospective unplanned subgroup analyses) are clearly indicated as such and deemed that unplanned analyses were appropriate and selective reporting would not appreciably bias results (e.g., appropriate analyses of an unexpected effect). This would include outcomes reported with insufficient detail such as only reporting that results were statistically significant (or not). |
| Probably High Risk of Bias (-) |
| - There is indirect evidence that all of the study’s measured outcomes (primary and secondary) outlined in the protocol, methods, abstract, and/or introduction, or mentioned in the discussion (that are relevant for the evaluation) have not been reported, - **OR** and there is indirect evidence that unplanned analyses were included that may appreciably bias results, - **OR** there is insufficient information provided about selective outcome reporting (record “NR” as basis for answer). |
| Definitely High Risk of Bias (--) |
| - There is direct evidence that all of the study’s measured outcomes (primary and secondary) outlined in the protocol, methods, abstract, and/or introduction (that are relevant for the evaluation) have not been reported. In addition to not reporting outcomes, this would include outcomes reported using measurements, analysis methods or subsets of the data that were not pre-specified or reporting outcomes not pre-specified, or that unplanned analyses were included that would appreciably bias results. |

- 1. *Other bias*

*11. Were statistical methods appropriate?*

We will consider the appropriateness of the statistical test undertaken. This is denoted by a yes/no answer using the dropdown box in the spreadsheet. Additional information will be provided stating why or why not the test is appropriate.

1. **References**

- Boase, J.; Ling, R. Measuring mobile phone use: Self-report versus log data. J Comput-Mediat Comm 2013;18:508-519
- Bowling, A. Mode of questionnaire administration can have serious effects on data quality. J Public Health (Oxf) 2005;27:281-291
- Catalogue of Bias Collaboration; Spencer, E.A.; Brassey, J. et al. Recall bias. in: Center for Evidence Based Medicine (CEMB), ed. Catalogue Of Bias (<https://www.catalogueofbiases.org/biases/recall-bias>); 2017
- Genaidy AM.; Lemasters GK.; Lockey J.; et al. An epidemiological appraisal instrument - a tool for evaluation of epidemiological studies. Ergonomics 2007; 50(6): 920-960
- Gerstman BB. Epidemiology kept simple 3rd ed., New York, NY: Wiley-Blackwell. 2013
- Higgins J.; Green S. Cochrane Handbook for Systematic Reviews of Interventions. The Cochrane Collaboration; 2011; Available: [www.cochrane-handbook.org](http://www.cochrane-handbook.org).
- Hutter, H.P.; Ehrenhofer, L.; Freuis, E. et al. Poor-to-moderate agreement between self and proxy interviews of mobile phone use. Bioelectromagnetics 2012;33:561-567
- Lauer, O.; Frei, P.; Gosselin, M.C. et al. Combining near- and far-field exposure for an organ-specific and whole-body RF-EMF proxy for epidemiological research: a reference case. Bioelectromagnetics 2013;34:366-374
- Mireku, M.O.; Mueller, W.; Fleming, C. et al. Total recall in the SCAMP cohort: Validation of self-reported mobile phone use in the smartphone era. Environ Res 2018;161:1-8
- NTP-OHAT. OHAT Risk of Bias Rating Tool for Human and Animal Studies. National Toxicology Program - Office of Health Assessment and Translation 2015
- NTP-OHAT. Handbook for Conducting a Literature-Based Health Assessment Using OHAT Approach for Systematic Review and Evidence Integration (March 4, 2019). National Toxicology Program - Office of Health Assessment and Translation 2019
- Parker, R.; Berman, N. Blinding in Observational Studies. In *Planning Clinical Research* (pp. 334-340). Cambridge: Cambridge University Press 2016. Available: doi:10.1017/CBO9781139024716.029
- Pettersson, D.; Mathiesen, T.; Prochazka, M. et al. Long-term mobile phone use and acoustic neuroma risk. Epidemiology 2014; 25:233-24
- Savitz, D.A.; Wellenius, G.A. eds. Interpreting Epidemiologic Evidence: Connecting Research to Applications. Oxford: Oxford University Press; 2016
- Toledano, M.B.; Auvinen, A.; Tettamanti, G. et al. An international prospective cohort study of mobile phone users and health (COSMOS): Factors affecting validity of self-reported mobile phone use. Int J Hyg Environ Health 2018;221:1-8
- Viswanathan M, Ansari M, Berkman ND, Chang S, Hartling L, McPheeters LM, Santaguida P.L.; Shamliyan T.; Singh K. et al. Assessing the risk of bias of individual studies when comparing medical interventions. Publication No. 12-EHC047-EF. Rockville, MD. Agency for Healthcare Research and Quality (AHRQ). Available: OHAT Risk of Bias Tool (January 2015) 2012; 37 <http://www.effectivehealthcare.ahrq.gov/index.cfm/search-for-guides-reviews-andreports/?pageaction=displayproduct&productid=998>.
- Whaley, P.; Aiassa, E.; Beausoleil, C. et al. Recommendations for the conduct of systematic reviews in toxicology and environmental health research (COSTER). Environ Int 2020;143
